# Paramedics’ Perspectives on the Hospital Transfers of Nursing Home Residents—A Qualitative Focus Group Study

**DOI:** 10.3390/ijerph17113778

**Published:** 2020-05-26

**Authors:** Alexandra Pulst, Alexander Maximilian Fassmer, Falk Hoffmann, Guido Schmiemann

**Affiliations:** 1 Department for Health Services Research, Institute of Public Health and Nursing Research, University of Bremen, Grazer Straße 4, 28359 Bremen, Germany; schmiemann@uni-bremen.de; 2 Health Sciences Bremen, University of Bremen, 28359 Bremen, Germany; 3 Department of Health Services Research, School VI - Medicine and Health Sciences, Carl von Ossietzky University of Oldenburg, Ammerländer Heerstraße 114-118, 26129 Oldenburg, Germany; alexander.fassmer@uni-oldenburg.de (A.M.F.); falk.hoffmann@uni-oldenburg.de (F.H.); 4 Department Institute for General Practice, Hannover Medical School, 30625 Hannover, Germany

**Keywords:** nursing home residents, hospitalization, hospital admission, patient transfer, referral, emergency department, emergency medical services, decision making

## Abstract

Emergency department (ED) visits and hospital admissions are common among nursing home residents (NHRs). Little is known about the perspectives of emergency medical services (EMS) which are responsible for hospital transports. The aim of this study was to explore paramedics’ experiences with transfers from nursing homes (NHs) and their ideas for possible interventions that can reduce transfers. We conducted three focus groups following a semi-structured question guide. The data were analyzed by content analysis using the software MAXQDA. In total, 18 paramedics (mean age: 33 years, male n = 14) participated in the study. Paramedics are faced with complex issues when transporting NHRs to hospital. They mainly reported on structural reasons (e.g., understaffing or lacking availability of physicians), which led to the initiation of an emergency call. Handovers were perceived as poorly organized because required transfer information (e.g., medication lists, advance directives (ADs)) were incomplete or nursing staff was insufficiently prepared. Hospital transfers were considered as (potentially) avoidable in case of urinary catheter complications, exsiccosis/infections and falls. Legal uncertainties among all involved professional groups (nurses, physicians, dispatchers, and paramedics) seemed to be a relevant trigger for hospital transfers. In paramedics’ point of view, emergency standards in NHs, trainings for nursing staff, the improvement of working conditions and legal conditions can reduce potentially avoidable hospital transfers from NHs.

## 1. Introduction

Nursing home residents (NHRs) represent a frail und vulnerable population group suffering from multimorbidity [1,2]. Changes or deteriorations in residents’ health status often result in hospital treatment. In several studies, the prevalence of hospital transfers from nursing homes (NHs) ranges from 6.8% to 45.7% for various time periods of follow-up [3]. In Germany, hospitalization rates of NHRs seemed to be higher than in other countries [4]. The risk of hospital admissions increases in the final months of life [5,6,7,8,9,10].

Health problems of NHRs are usually managed by a general practitioner (GP) or other medical specialists. In contrast to other countries, physicians in Germany are not directly employed by NHs. Symptoms that occur in the evening, night or at weekend can be managed by outpatient out-of-hours medical care (OOHC). In Germany, this service is provided by the associations of statutory health insurance (SHI) physicians and can be reached via a central telephone number (call 116 117). The OOHC provides telephone support, walk-in clinic as well as home visits [11]. In contrast to the OOHC, the emergency medical services (EMS) (call 112) is primary responsible for the initial treatment and transport of patients with urgent and life-threatening conditions [12]. Both care systems are organized independently from each other. However, interventions performed by paramedics and emergency physicians increased in NHs in the last years [13,14]. In Germany, every twelfth EMS transport (8.3%) each year is requested to NHs [15]. Hospital transfers of NHRs were often initiated by nurses without involvement of a physician [16]. Due to demographic changes, EMS utilization might increase in future [17] contributing to increased workload in the emergency departments (EDs) [18].

Several studies indicated that many ED visits and hospital admissions from NHRs are inappropriate or avoidable [19] and can increase the risk of iatrogenic diseases and delirium [20,21].

As a clear definition of these terms is lacking, the proportion ranges between 1.7% and 67% depending on definition and study design [22,23,24]. Most studies in this research area are based on analysis of ambulatory care sensitive (ACS) conditions [25,26,27] or represent the perspectives of GPs [28,29,30] or nursing staff [16,31]. Research on the perspective of paramedics who are involved in the hospital transfer process of NHRs on a daily basis is scarce. The authors of these two existing studies [32,33] mainly described the insufficient documentation of residents’ information and issues to understand and consider residents’ wishes. However, so far, there is no study existing which determined these paramedics’ experiences in the context of health care conditions in Germany.

In the German project *HOspitalisations and eMERrgency department visits of Nursing home residents* (HOMERN) we examined the health care of NHRs with a special focus on hospital transfers. The project used several data sources and methods like statutory health insurance data [34], questionnaire surveys with physicians and nursing managers [35] and cross-sectional data of NHs in Germany to analyze reasons for hospitalization. To gain further perspectives on this topic, this study aimed to explore how paramedics experience the transfer process of NHRs, generating ideas for interventions that aim to reduce hospital transfers in this setting.

## 2. Materials and Methods

This study used a qualitative approach to answer the research question. We chose focus groups to gain new insights into a complex topic gathering in-depth data of this professional group regarding the transfer process. During the planning of the focus groups, we followed methodological guidelines [36,37,38] on recruiting, moderation and conducting. Ethical approval for this study was given by the ethics committee of the medical association in Bremen, Germany (application number 613/A). This study followed the consolidated criteria for reporting qualitative research (COREQ) [39].

### 2.1. Recruitment and Study Population

Snowball sampling was applied to recruit participants for the focus groups. We contacted regional EMS organizations and used existing cooperation to NHs, personal and professional contacts, their networks, and social media to identify potentially interested persons. An invitation letter containing the purpose of the study, date and place of focus groups and contact information was sent via email. Participants were included if they (1) were emergency medical technicians (EMTs) (“Rettungssanitäter”), paramedics (“Rettungsassistenten”) or emergency paramedics (“Notfallsanitäter”) (paramedics with higher level of certification and extended competences since 2014 in Germany), (2) had experience with emergency care of NHRs and (3) worked in Bremen or surrounding regions of Lower Saxony in Germany. Because EMS in Germany are structured and organized differently, we intended to address persons of different EMS sponsorships, regions (rural, semiurban, urban) and levels of working experience to reach a sample of maximal variation. Persons willing to participate received a confirmation letter thanking for interest and providing additional information about planning. Participants were reminded few days before via telephone. Recruitment was coordinated by two researchers (AP and GS) who also participated in the focus groups.

### 2.2. Focus Group Process

We conducted the focus groups with paramedics between April and June 2019 in Bremen, Germany. The aim was to identify paramedics’ experiences in the cooperation with NHs and possible needs for action to reduce potentially avoidable hospital transfers of NHRs. As recommended for focus groups [37], we planned at least three group discussions. The discussions took place in rooms of the Association of SHI Physicians in Bremen. The discussions lasted between 112 and 125 min and were audio-recorded. Each group was moderated by AP (researcher, female) and was assisted by GS (researcher, private lecturer and GP, male) and a student worker (female) who took notes and documented the session. Both researchers (AP and GS) had backgrounds in health services and nursing research for several years and were engaged in the HOMERN project.

We used a semi-structured question guide (see Supplementary Materials Table S1) to structure the discussions. The guide was based on existing literature and discussed with project group members beforehand. We followed the guidelines of Krueger (1997) to develop appropriate open-ended questions [40]. The interview guide was pilot tested previously with one paramedic who was recruited through personal contact of one researcher (AP). Due to his comments, we changed the order of questions and considered organizational aspects for planning of the focus groups.

After the first focus group, the guide was slightly modified to improve data content without changing consistency. Prior to the group discussions, the participants were informed about purposive/reason of the study, researchers’ interest in the research topic and information that participation was voluntary, and responses would be anonymized. In addition, a short questionnaire on sociodemographic data (sex, age, professional group, and years of working experience) was completed. Participants gave written informed consent to the study before sessions started.

The discussions started with a brief introduction of each participant about their general experience with NHs in their daily work. Participants were requested to take notes on moderation cards that were clustered on a flipchart. Headed by the chronology of the transfer process, the following topics were addressed: the initiation of, and reasons for, an emergency call (“112”), experiences at arrival in NHs, and influencing factors on the transfer process (e.g., advance directives (ADs), role of relatives). Across these topics, we focused mainly on three exploratory questions: “Which experiences do paramedics perceive in cooperation with NHs?”, “How would paramedics define an ‘avoidable’ hospital transfer among NHRs?” and “Which interventions would paramedics suggest to reduce hospital transfers?” (see Supplementary Materials Table S1). Because a specific definition of ‘avoidability’ is missing, we used an open question to explore new insights on paramedics’ perspective. At the end of the sessions, both researchers summarized key themes and participants were asked to confirm researchers’ summary. After the third focus group, we reached data saturation, additional data did not gain any further information or new understandings.

Each participant received a financial allowance of 50 euro for participation (compensation for time and transport) in the study. The audio recordings were transcribed by professional transcriptionist office, anonymized and added by notes which were documented during focus group discussions.

### 2.3. Data Analysis

We used qualitative content analysis by Elo and Kyngäs to identify common perspectives of paramedics with transfer process of NHRs [41]. Analysis was oriented by our focus group guide and was complemented by new topics which emerged during focus groups discussions (e.g., legal conditions or ethical aspects). We therefore combined a deductive and inductive approach. Firstly, two researchers (AP and AF) independently read transcripts several times to get an overview and get familiar with data (preparation phase). AP used open coding by writing notes and headings while reading transcripts until all aspects of content were described. A coding sheet was developed to group and order headings into categories and subcategories. Coding sheet and coding results were controlled and reviewed by AF. Disagreements were discussed between AP and AF until consensus was reached. The transcripts were then analyzed again (organizing phase). We followed the chronology of the transfer process to structure data analysis (reporting). The consensus coding process was supported by the software MAXQDA Analytics Pro 2018. To cross-check our findings, we summarized the results of all three groups and returned them to the participants via email. According to the COREQ requirements [39], participants were invited to comment if all relevant contents were covered by the main themes and if they would modify themes (participant checking). After two weeks, 5 of 18 participants responded to the request and gave feedback via email. As a result, no major revisions emerged.

## 3. Results

### 3.1. Characteristics of Participants

The total sample consisted of n = 18 participants (n = 14 male; n = 4 female) across three focus groups. They were aged between 21 and 55 years (mean: 33 years). N = 4 participants worked as EMTs, n = 4 were paramedics and n = 10 were emergency paramedics. Working experience ranged between one and 29 years (mean: 10 years). In total, 11 of 18 (61%) participants worked in urban areas (>100,000 residents) and 7 of 18 (39%) worked in semi-urban (>20,000 and ≤100,000 residents) or rural areas (≤20,000 residents) in the region around the city of Bremen in Germany. A detailed description of focus group members is illustrated in Table 1.

**Figure 1:**
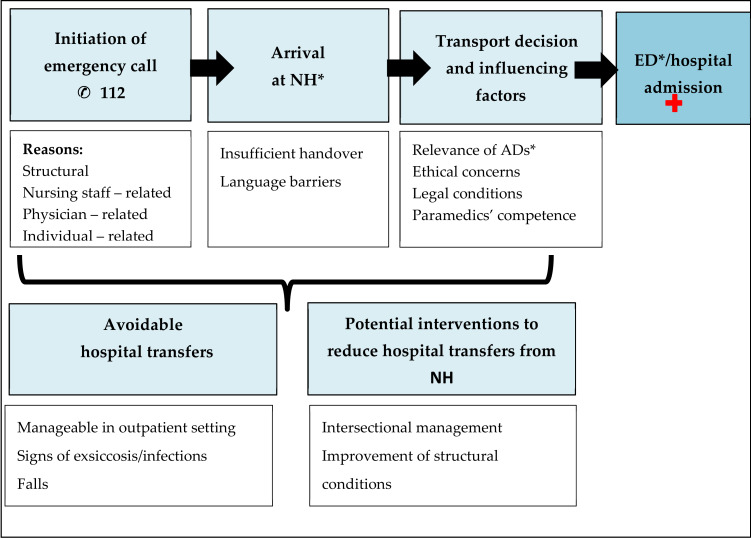
Main themes in the hospital transfer process: an overview of categories and subcategories. * AD: advance directive, ED: emergency department, NH: nursing home.

Five main themes emerged during focus groups and could be classified in chronological order of transfer process: “initiation of emergency call”, “arrival at NH”, “transport decision and influencing factors”, “avoidable hospital transfers” and “potential interventions to reduce hospital transfers from NH” (see Figure 1).

### 3.2. Initiation of Emergency Call

#### 3.2.1. Structural Reasons

When an emergency call (“112”) was initiated, the decision to send out the EMS was taken by the dispatcher in the ambulance control center. Paramedics reported that dispatchers were personally liable for all decisions they made. Even though an emergency call was initiated because of non-serious symptoms, the dispatcher would always send out an ambulance to be on the safe side. Already at this point of the transfer process, avoidable hospital transfers may be identified but could not be prevented:

*[…] it is incredibly difficult for the dispatchers on the phone to decide […] what is going on on-site […] it is acute, it is not acute […]. It is more difficult to find out if it’s just this or if medical assessment is needed. (B5, FG1)*

The dispatcher is also responsible for deciding which kind of transfer is necessary. In Germany, there is a main difference between (a) patient transports for planned or non-urgent cases that require a prior medical order by a physician and (b) emergency rescue transports that can be carried out without a prior medical order. Even though a large number of NHRs could be transferred in the form of a planned patient transport, this does not happen:

*[…] [if] there was no physician on-site and did not fill out any transport order before, then the dispatcher had to send out an ambulance […]. He does not have to say: “You do not get an ambulance, organize the transport order firstly” or something like this. Instead, they are rigorously sending out an ambulance. And then, we are standing there again (laughing). (B2, FG2)*

Paramedics observed difficult working conditions in NHs. From their point of view, staff shortage and lack of time were main reasons for the initiation of an emergency call:

*There is too few staff in the NHs, they cannot manage their work at all. Actually, they only try to handle these things which are very very important, everything else is actually not possible anymore. (B3, FG2)*

*I think that if we say: “Please monitor the patient”, they know that they can’t monitor the patient adequately because they do not have time for it. (B5, FG2)*

As a result of these working conditions, nurses would not be able to manage care of NHRs adequately. Especially at night, on weekends and before holidays, paramedics had the impression that nurses tried to get rid of some residents:

*It feels like if it is easy for the staff to get rid of the residents, they simply cannot manage it anymore. Something I could understand. They have too much patients there. (B2, FG2)*

#### 3.2.2. Nursing Staff-Related Reasons

On the one hand, paramedics felt sorry for nursing staffs’ working conditions and admitted that there was no personal blame on them. On the other hand, they still criticized nurses’ behavior of calling the ambulance. Paramedics traced this back on insufficient professional nursing expertise:

*[…] there are often only bagatelles why we are called. This can be just a little infection or fever and then they are like overstrained. And they do not know what to do, because they simply do not have the qualification […]. (B1, FG3)*

*[…] well I think, they should know the all medication prescribed and their indication when they are giving them their residents every day. This would make life easier. (B2, FG1)*

In addition, paramedics perceived nurses’ willingness to assume responsibility as poor. They would often prefer the EMS instead of first managing the problem in NHs and by themselves:

*[…] well, somehow this is the greatest issue and it occurs actually during every drive in NHs. „I just want to be on the safe side.“ […] For example, there is a big discussion in one NH, they send every patient to the hospital who fell. Because he could have a brain hemorrhage […]. And if we discuss with them endlessly, they insist on a transfer. (B2, FG1)*

Paramedics questioned this behavior, especially when a resident was competent to give consent and the decision for an emergency call was made without resident’s involvement.

#### 3.2.3. Physician-Related Reasons

Besides staff shortage and lack of time, nurses’ behavior was assumed to be a reaction on insufficient cooperation with GPs or other physicians:

*[…] there is mostly no communication with the GP […]. That is also a big problem. He could equally move out to look after his patients, to prescribe something and all residents could remain in the NH. (B3, FG2)*

Paramedics reported that physicians were sometimes not able to visit NHRs personally. As a result, nurses were forced to contact the EMS to manage the situation:

*[…] the resident does not feel well, and the nursing staff has also other residents to care for. So, they have to find a solution. That means, they call the GP […] The GP says: ‘I cannot move out’. In the next instance they think – ‘Okay, if the GP cannot move out. I am afraid of that the resident gets worse and I could get into trouble, therefore I will call the EMS’. (B5, FG1)*

*[…] the GP is not available or cannot move out. The GP/OOHC or whoever is responsible in this case. It occurs often enough that the EMS is called as a substitute for the physician. (B6, FG3)*

In many cases, physicians could not be reached by telephone. As a consequence, nurses had to make decisions regarding patient transfer on their own. This resulted in emergency calls without the involvement of the physician.

#### 3.2.4. Individual-Related Reasons

Apart from the interests of nursing staff and physicians, emergency calls were, in some cases, initiated because of pressure from family members.

*Of course, this is an aspect which we often hear from the nursing staff. ‘The relatives put pressure on me if I will not do this or that’. The claims of relatives increased enormous. (B2, FG2)*

*[…] if the patient says: ‘I want to be transported’, or if the relative which has the power of attorney says: ‘the patient has to be transported’, I cannot not transport him. (B5, FG1)*

### 3.3. Arrival at Nursing Home

Paramedics reported about insufficient handover when arriving in NHs. In some cases, a lot of time was wasted because nobody waited for paramedics and briefed them about the reason for calling the EMS:

*[…] lacking responsibility. I often experience it when I come to the ward of a NH. It begins, for example, right at the entry. The care manager or anybody else does even not know that the EMS was called, or on which ward we have to get. Of course, this does not always happen, but it gets more frequent. (B2, FG1)*

*[…] I often notice that the ambulance was called and then there is nobody when we arrive. Then we do not know on which floor and which ward we have to get because nobody is waiting for us […]. (B2, FG3)*

From their point of view, these situations can also be traced back to staff shortage in NHs. Nevertheless, paramedics criticized nurses’ insufficient preparation regarding medical and organizational issues and lack of information:

*[…] It is no secret that the ambulance is coming, […] because normally the nursing staff called us. […] And then, the patient history and medication plans are not available. […] This is time extensive, especially in really urgent cases. It would be nice, if we can take a look on resident’s prior history, ADs and medication plans at least. In an acute situation it is meaningful to have this information close at hand […]. (B2, FG3)*

In this context, paramedics sometimes experienced communication problems because of language barriers. Moreover, during handover, ADs were required, but paramedics underlined that they were often missing/not available in NHs:

*[…] ADs are a difficult story. Not everyone has one. And if ADs are existent, you have to look for them firstly. And maybe they are not in the NH but at the daughter’s home […] and so on. (B2, FG1)*

### 3.4. Transport Decision and Influencing Factors

When ADs were available, their relevance to a transfer decision is low. Instead, paramedics underlined their importance in case of life-threatening situations and for further treatment in the hospital. The transport decision was accompanied by ethical concerns, especially when palliative residents in end-of-life situations or residents with dementia were exposed to stressful situations in the ambulance car and overcrowded ED:

*[…] we often experienced that. With patients who have dementia or with very old and fearful residents. […] the transport is horrifying for them. It is shaking in the ambulance and they go to the hospital. I often heard in this context: ‘Oh my god, they will never let me out again’ [...], they are frightened. (B3, FG1)*

*We also transport palliative patients which is not necessary. They are going to hospital to die there […]. They need oxygen, they need calm, they maybe need someone who wet their lips. Analgesics. But they do not need a hospital. (B3, FG2)*

In some cases, paramedics did not understand the need for emergency calls. Even if they considered transfers as not necessary because of harmless symptoms, the legal conditions in Germany forced them to transport each resident to the hospital:

*We arrive at the NH and see that the resident is in better hands on-site. But we are no physicians. In Germany just a physician is allowed to make a diagnosis. Therefore, we cannot keep the resident in the NH. (B5, FG1)*

*We have clear instructions. We have the obligation to transport. We arrive, take the patient with us. This is our job unless the patient refuses. (B6, FG1)*

Most paramedics attempted to prevent avoidable hospital transfers and tried to discuss further treatment. If NHRs were capable and gave written consent to refuse transport, they could remain in the NH. In case of dementia, paramedics contacted family members to come to an agreement. They also seek for other alternatives in outpatient care:

*[…] just because we leave someone in the NH, it does not mean that we do not send him other medical support. […] We have also the possibility […] to call the OOHC, to describe the situation and to hear how they assess it. Or we call the emergency physician […] to get a solution on-site […] and to keep the resident in his familiar environment […]. (B3, FG1)*

Some paramedics describe, in this context, that these kinds of reassurances were limited when physicians were not available or because the ambulances were not equipped with mobile phones to call for feedback. In these cases, they were forced to operate in a legal grey area.

### 3.5. Avoidable Hospital Transfers

Being asked which hospital transfers might be avoidable, paramedics primarily referred to clinical symptoms that could be managed in an outpatient setting. Lots of avoidable transports were carried out because of catheter problems. Even though registered nurses in Germany are trained in placing and changing catheters, this did not happen often due to internal instructions and the kind of catheter. When urologists and GPs were not available, nurses called the ambulance. Paramedics reported that these transports were frequent and therefore time and resource intensive. Similar problems were discussed in the context of ruling out fractures after minor trauma (e.g., falls). Further avoidable transfers, for example, because of exsiccosis or infections, were based on too little time for close monitoring of symptoms:

*A patient with a feverish infection or an urinary tract infection does not need to be transferred to hospital by an ambulance. […] I think […] that is a combination of […] medical care, nursing competence and […] the physicians are not really available and there is a lack of nursing staff resources to monitor the situation closely. (B2, FG3)*

### 3.6. Potential Interventions to Reduce Hospital Transfers from NHs

From the perspective of paramedics, avoidable hospital transfers were mainly caused by structural deficits and difficult working conditions in NHs. Essentially, they saw a need for political action regarding staffing ratio and financial compensation, which could improve the image of nursing profession. While discussing specific approaches to reduce hospital transfers, the use of more resources in outpatient care was suggested. Paramedics recommended to interconnect the phone lines of EMS (“112”) and OOHC (in Germany “116 117”). Nurses, who call the EMS in non-serious cases, could be referred to an outpatient physician via telephone. This option was also discussed as a useful resource for paramedics if further medical support was needed. Another telephone resource was discussed as a solution to resolve legal uncertainties. Paramedics wished to get reinsurance for need of transport via telephone or telemedicine services:

*There is simply an instance missing on which I can reinsure myself. Someone to whom I can say: ‘I have this and those things, did I overlook something?’ And he says: ‘No, you can keep this patient with clear conscience in the NH.’ Then we keep him there or ‘No, you have to consider this and that, it should be clarified clinically.’ (B5, FG1)*

*And there has to be a point of contact, not for general public but for medical professionals - emergency paramedics, nurses in NHs, nurses in outpatient care […] who can contact a person who is just responsible to resolve these issues. (B5, FG1)*

On the level of NHs, interventions focusing on working processes may be helpful. Paramedics discussed in this context the introduction of emergency standards to define when the EMS is needed and in which cases the OOHC is sufficient. These would guide nurses in case of acute health changes and could improve quality of handover:

*Well, it would be nice if they all had the same standards like in emergency care. […] what to do in case of an emergency and what are the most important things […]. It does not need to be a big program, they also do not need to learn how to insert a venous access. But they should simply know that an open door is good for us, for example. Or that the bedside table has to be put away. These kinds of stories. (B6, FG1)*

Further approaches to reduce hospital transfers from NHs might aim on qualification and competences. Paramedics appreciate the idea of an NH physician, which can ensure that all residents of one NH are regularly visited. However, this profession is not established in Germany so far. Instead, minor health problems could be managed without the involvement of the physician or EMS when nurses were qualified (e.g., regarding catheter problems or signs of exsiccosis). In addition, paramedics recommended training for nurses regarding emergency standards, the relevance of ADs and the communication of residents’ wishes, which may be integrated in nursing education. To reduce uncertain behavior, information relating to legal and medical aspects of care would be needed. Paramedics considered exchange with nurses as very important to develop two-ways of understanding for each profession:

*[…] it would be certainly helpful for them to do an internship in the EMS. […] To see which problems we get if documentation in the NH is not available or incomplete. […] Maybe they would develop another view if they go with us on the ambulance. Or if we simply get together. (B2, FG2)*

## 4. Discussion

### 4.1. Issues during the Transfer Process

This qualitative study explored experiences of paramedics with hospital transfers from NHs. Our results suggest that structural reasons are relevant contributors to avoidable ambulance calls. Paramedics often felt that they were called as a substitute for the GPs, as they perceived nursing staff to be unable to handle every health change due to high workload and insufficient visits from physicians. However, required transfer information (e.g., residents’ wishes, medication plan) was missing or not complete when paramedics arrived at NHs. Responsible nurses who can give reliable information about resident’s condition were perceived as scarce. These challenges of poorly organized handovers were also reported in other studies [33,42]. Jones et al. showed in this context that nearly 10% of NHRs were transported to hospital without any documentation [43]. ED physicians considered this insufficient information as difficult for further and rapid treatment in hospital [33].

A study of Murphy-Jones et al. is, so far, the only one that examined paramedics’ perspectives on the transfer process of NHRs, focusing on end-of-life care. According to these results, paramedics’ difficulties to understand and evaluate residents’ preferences are due to poor documentation as well as insufficient information and the qualification of nursing staff [32]. In our study, we could verify these findings. However, we could identify further issues at the whole process from initiation of an ambulance call to the final transport. We showed that uncertainties may not only affect nursing staff as already known from other studies [44,45,46]. Instead, it seems to be that all involved health care providers (physicians, dispatchers, and paramedics) fear legal consequences of a non-transport. This can lead to negative interactions between the professional groups, for example, when paramedics were called in case of harmless symptoms that did not justify the use on an ambulance in their point of view. In these situations, they traced transfers back to insufficient nursing expertise. In a study by Jablonski et al., nursing staff confirmed these interactions and reported that they felt they were not being taken seriously by paramedics and instead were perceived as overreacting [47]. Some paramedics reported in this context to feel also pressured by family members or note this pressure on nursing staff. This influence of family members was also shown in a recent systematic review [48]. It seemed to be that the different views of these involved groups were a challenge during the transfer process. Physicians who should decide about further treatment were often lacking in these situations so that paramedics were forced to transport every resident for medical assessment.

In some cases, ambulance calls caused ethical considerations in paramedics. Besides the fact that transfers in case of harmless symptoms might have a negative impact on residents’ quality of life [32], many time and personnel resources of the EMS were lacking for patients with potential life-threatening symptoms. Paramedics questioned the benefit of hospital transports, especially in palliative cases. ADs may be helpful to keep NHRs on-site, but these were not always available or valid. Issues of unknown resident’ wishes were also found in other studies [49,50]. Considering a dementia prevalence of 45–62% among NHRs [51,52,53], paramedics might be often faced with this problem during transfer process. Deficits were also reported by GPs [54] and medical directors/directors of nursing [55] assuming that improved information around end-of-life care and communication of NHRs’ wishes between all involved groups might reduce hospital transfers. However, adequate advance care planning might be just one of several required interventions because “Do-Not-Hospitalize Orders” solely cannot guarantee appropriate treatment in NHs as a substitute for a hospital stay [56].

### 4.2. Avoidable Hospital Transfers

Paramedics perceived the hospital transfers of NHRs as avoidable in case of catheter problems, exsiccosis, infections and in some kind of falls. They suggested that these cases are time consuming and cost intensive and could be usually managed in an outpatient setting. This is consistent with the perspectives of physicians reported by Ouslander et al. This suggests that paramedics may be able to assess minor illnesses or symptoms on-site, but have no possibilities to reduce these transfers due to legal conditions and pressure from others. Further reasons that contribute to avoidable transfers are the insufficient care of physicians, poor management of ADs, NHRs’ low benefit of a transfer and pressure of family members. These aspects were mentioned by physicians [57,58] and nursing staff equally [16,31,59] and can also be found in our study. Even if our study did not primarily focus on the avoidability of transfers, our findings show that paramedics’ views were consistent with other involved professional groups. This may be helpful in the development of interventions reducing avoidable transfers from NHs.

### 4.3. Approaches to Reduce Hospital Transfers

Due to legal uncertainties in the decision-making process, the majority of hospital transfers can just be reduced by complex political interventions (e.g., improving nursing staff ratio or legal conditions). Some interventions that were suggested by paramedics are currently pilot tested in Germany. For example, some regions interconnected the European emergency call number “112” and the number “116 117” for OOHC to coordinate more harmless health problems into outpatient structures [60]. The effects of this intervention are unknown as they started in early 2020 and have not been evaluated yet. Another current pilot project aimed on outpatient treatment of non-life threatening patients by specialized community paramedics (“Gemeindenotfallsanitäter”) [61]. These paramedics were already dispatched in rescue directing centers and sent out to patients who do not require immediate emergency care. In contrast to “regular” paramedics, community paramedics received special training and more medical competences, for example, to change catheters. This intervention might have potential to avoid hospital transfers, as already shown in other projects [62]. Effects of the intervention in Germany will be evaluated after the end of the project in 2021.

Besides structural conditions, paramedics saw need for the better communication of resident-related information during the transfer process. In this context, studies showed that information gaps for NHRs transferred to hospital were common [43,63]. Insufficient information was mainly related to reasons for transfer, baseline cognitive function and communication ability, vital signs, ADs and medication [63]. ED physicians perceived the insufficient information as difficult for further and rapid treatment in hospital [33]. Within our study paramedics discussed a nationwide implementation of obligatory emergency standards in NHs. These could help nursing staff to decide in which cases an emergency call is appropriate and to ensure which processes and information has to be prepared before paramedics arrive in NHs. This idea was also discussed in other studies [33,64]. Standardized transfer forms—as one part of these standards—were already developed in some countries [65,66]; however, the use of them is just recommended and not obligatory. For a successful implementation of standards, qualified nursing staff is needed. Paramedics in our study criticized inadequate handovers because nurses were often not prepared and not able to give report about residents pre-existing conditions and symptoms. This perceived lack of qualification could only be improved by structural interventions. These might include (emergency) trainings for nursing staff and internships in emergency services during nursing education. Furthermore, the specialization of nurses is feasible to decrease hospitalization, for example, in case of catheter problems or palliative cases. Studies showed in this context, that specialized “nurse practitioner”, “physicians assistants” or “advance practice nurses” [67,68] can improve health care and decrease hospitalization of NHRs [69,70].

### 4.4. Strengths and Limitations

Research on paramedics’ view on transfer process from NHs is scarce. We closed this gap in research and provided evidence based on conditions of the German health care system. In contrast to the only qualitative study with paramedics [32], we used a bigger sample to generate data. We included EMTs, paramedics and emergency paramedics to represent all professional groups of EMS. Even though EMTs were not presented in one focus group, this may not affect our results due to their lower competences and limited permission regarding decision making in Germany. Referring to the trustworthiness criteria of Lincoln and Guba [71], we aimed to increase confirmability by using general and open questions to prevent researcher bias during discussions. However, we cannot exclude selection effects due to the payment of financial allowance. This possibly influenced the willingness of participants and the dependability of the results. To ensure representative findings, we included participants which differed in age, sex, region and sponsorship of EMS. The focus group participants checked our findings to improve the credibility of the results. We followed this method, as also recommended by the COREQ guidelines for qualitative research [39]. Due to the organizational differences between EMS, the transferability of our findings to other regions or countries might be limited.

## 5. Conclusions

The decision-making process of hospital transfers is complex, and paramedics are faced with several issues in the cooperation with NHs. Uncertainties and the fear of legal consequences seem to be the main trigger and affect all involved groups (nursing staff, physicians, dispatchers, and paramedics,). Due to the working conditions in NHs, which are characterized by a lack of time and limited contact with physicians, paramedics perceived the required information during handover as insufficient. In particular, medication plans and ADs were often incomplete or missing. As medical diagnoses are in the responsibility of physicians who often are not available, paramedics have to carry out every transport even if they rated them as avoidable (e.g., catheter problems or infections). Their influence to reduce transfers is therefore low. With this study, we closed a gap in research, providing the first qualitative study in Germany exploring hospital transfer process of NHRs from the perspective of paramedics. Our results suggest that political long-term interventions are required to improve structural and legal conditions in Germany. Short-term interventions that may reduce the transfers of NHRs should focus on emergency standards in NHs and improved communication between nursing staff and paramedics, for example, in the form of specialized education and emergency training.

## Figures and Tables

**Table 1:** Description of focus group members.

Focus Group	Participants (n)	Sex	Age (Years)	Profession	Working Experience (Years)	Working Area
1	7	Male n = 6 Female n = 1	Range: 21–44 Mean: 31	EMT * n = 2 Paramedic n = 2 Emergency paramedic n = 3	Range: 3–23 Mean: 7	Urban n = 4 Semi- urban/rural n = 3
2	5	Male n = 4 Female n = 1	Range: 26–34 Mean: 29	Paramedic n = 1 Emergency paramedic n = 4	Range: 2–14 Mean: 8	Urban n = 4 Semi- urban/rural n = 1
3	6	Male n = 5 Female n = 1	Range: 23–55 Mean: 28	EMT * n = 2 Paramedic n = 1 Emergency paramedic n = 3	Range: 1–29 Mean: 13	Urban n = 3 Semi- urban/rural n = 3

* EMT: emergency medical technicians.

## Supplementary Materials

The following are available online at https://www.mdpi.com/1660-4601/17/11/3778/s1. Table S1: Focus group question guide.

## Abbreviations

ACS	ambulatory care sensitive
AD	advance directive
COREQ	consolidated criteria for reporting qualitative research
ED	emergency department
EMS	emergency medical services
EMT	emergency medical technician
GP	general practitioner
NH	nursing home
NHR	nursing home resident
OOHC	out-of-hours medical care
SHI	statutory health insurance
