# Supplementary material for: Paramedics’ Perspectives on the Hospital Transfers of Nursing Home Residents—A Qualitative Focus Group Study
Source: Int J Environ Res Public Health. 2020 May 26;17(11):3778. doi: 10.3390/ijerph17113778 (PMC7312002; doi:10.3390/ijerph17113778)
Supplement: Supplementary file 1 [file ijerph-17-03778-s001.pdf]

Supplementary material

Table S1: Focus group question guide

Opening question at the beginning to collect and cluster thoughts on moderation cards:<br><br>**Which aspects come to your mind thinking about your everyday experiences in NHs?**<br><br>**Prompts:**<br>Which experiences do you perceive in cooperation with NHs?<br>What are your impressions when you arrive in NHs?<br>How are transfer decision made? What are influencing factors related to transfer decisions? Who is involved?
**How would you define an "avoidable" hospital transfer among NHRs?**
**Which interventions would you suggest to reduce hospital transfers from NHs?**
